# IL6 secreted by Ewing sarcoma tumor microenvironment confers anti-apoptotic and cell-disseminating paracrine responses in Ewing sarcoma cells

**DOI:** 10.1186/s12885-015-1564-7

**Published:** 2015-07-28

**Authors:** Andrej Lissat, Mandy Joerschke, Dheeraj A. Shinde, Till Braunschweig, Angelina Meier, Anna Makowska, Rachel Bortnick, Philipp Henneke, Georg Herget, Thomas A. Gorr, Udo Kontny

**Affiliations:** 1 Division of Pediatric Hematology and Oncology, Charité – University Medical Center, Berlin, Germany; 2 Division of Pediatric Hematology and Oncology, Department of Pediatrics and Adolescent Medicine, University Medical Center Freiburg, Freiburg, Germany; 3 Dheeraj Shinde, Institute of Oncology Research, Via Vincenzo Vela, Bellinzona, 66500 Switzerland; 4 Institute of Pathology, RWTH Aachen University, Aachen, Germany; 5 Center for Chronic Immunodeficiency, University Medical Center Freiburg, Freiburg, Germany; 6 Department of Traumatology and Orthopaedics, University Medical Center Freiburg, Freiburg, Germany; 7 Institute of Veterinary Physiology, Vetsuisse Faculty, University of Zurich, Zurich, Switzerland; 8 Division of Pediatric Hematology and Oncology, University Medical Center Aachen, Pauwelsstraße 30, Aachen, 52074 Germany

**Keywords:** Ewing sarcoma, Tumor microenvironment, IL6, Migration, Apoptosis

## Abstract

**Background:**

The prognosis of patients with Ewing sarcoma (ES) has improved over the course of the last decades. However, those patients suffering from metastatic and recurrent ES still have only poor chances of survival and require new therapeutic approaches. Interleukin-6 (IL6) is a pleiotropic cytokine expressed by immune cells and a great variety of cancer cells. It induces inflammatory responses, enhances proliferation and inhibits apoptosis in cancer cells, thereby promoting chemoresistance.

**Methods:**

We investigated expression of IL6, its receptors and the IL6 signal transduction pathway in ES tumor samples and cell lines applying reverse transcriptase PCR, immunoblot and immunohistochemistry. The impact of IL6 on cell viability and apoptosis in ES cell lines was analyzed by MTT and propidium iodide staining, migration assessed by chorioallantoic membrane (CAM) assay.

**Results:**

Immunohistochemistry proved IL6 expression in the stroma of ES tumor samples. IL6 receptor subunits IL6R and IL6ST were expressed on the surface of ES cells. Treatment of ES cells with rhIL6 resulted in phosphorylation of STAT3. rhIL6 protected ES cells from serum starvation-induced apoptosis and promoted migration. IL6 blood serum levels were elevated in a subgroup of ES patients with poor prognosis.

**Conclusions:**

These data suggest that IL6 contributes to ES tumor progression by increasing resistance to apoptosis in conditions of cellular stress, such as serum starvation, and by promotion of metastasis.

**Electronic supplementary material:**

The online version of this article (doi:10.1186/s12885-015-1564-7) contains supplementary material, which is available to authorized users.

## Background

Over the course of the last 20 years survival rates for patients with Ewing sarcoma have increased as a consequence of therapy intensification, improvement of surgical techniques and radiotherapy. However, even with multimodal therapy regimens including intensive chemotherapy and interval compression 5-year EFS for patients with ES is around 70 % [1, 2]. In recent years, a multitude of new insights into the biology of ES cells has been made allowing the introduction and development of targeted ES therapies [3]. In particular, patients suffering from metastatic and recurrent disease with long term overall survival rates of only around 20% are in need of such new therapeutic approaches [4].

Genetic alterations serve as the basis for disturbed signaling pathways. Analysis of gene expression data from ES cell lines after inhibition of EWS-FLI1 expression demonstrated that several mediators of inflammation, including SOCS3, IL6ST, IL-1-accessory protein and IL-8 are regulated by EWS-FLI1 [5]. Inflammation plays an important role in the development of a great variety of tumors (reviewed in [6, 7]). In terms of the clinical presentation of ES, it is well known that the distinction between osteomyelitis and malignancy can often be made via biopsy only [8]. In line with this, patients suffering from ES often present with fever, itself indicating a worse prognosis [9]. Moreover, elevated IL6 levels in peripheral blood of patients with bone sarcomas correlate with higher tumor extension and decreased overall survival [10].

In other malignant diseases, such as Hodgkin’s lymphoma or neuroblastoma, high IL6 blood serum expression is associated with poor prognosis [11, 12]. In multiple myeloma cells IL6 inhibits apoptosis from dexamethasone, serum starvation and FAS-ligand [13]. In neuroblastoma IL6 protects cells from drug-induced apoptosis and increases proliferation [14]. Tumor cells derived from epithelial tissues like mammary, prostate and colon carcinoma show increased IL6-induced expression of BCL-X_L_ [15, 16], Hsp70 [16], cyclin A1 [17], and Mcl1 [18]. In addition to inhibiting apoptosis, IL6 signaling is also known to promote migration and invasion in a great variety of tumors including ovarian cancer, breast cancer, glioblastoma and chondrosarcoma [19–22].

The biological effects of IL6 are mediated via the IL6 receptor complex consisting of IL6ST, the signal transducing component expressed in a great variety of human tissues, and the specific IL6 binding subunit IL6R, which is found membrane-bound on the cell surface and soluble in the sera of patients. The soluble IL6R subunit mediates IL6-trans-signaling through formation of an IL6/IL6R complex, which interacts with membrane bound IL6ST. This permits IL6 signaling even in those cells which do not express IL6R [23, 24]. Examination of the complex interactions of IL6, IL6R and IL6ST by Boulanger et al. revealed that interaction of IL6 and IL6R is followed by tertiary and quaternary changes of protein structure alleviating binding to IL6ST. The result is transition to a hexamer which is highly competent to transduce the extracellular signal [25]. The main signaling pathways activated by IL6 are the STAT3- and MAPK-pathways leading to altered gene expression, which among others comprise genes related to metastasis, apoptosis and proliferation [26].

Since inflammation plays a significant role in the clinical presentation of ES and elevated IL6 levels - associated with poor prognosis - are found in ES patients, we have analyzed the expression and functionality of the IL6/IL6R system in ES.

## Methods

### Patients and tissue samples

All but one tumor specimen and all serum samples used in this study were obtained at the time of initial diagnosis; for one patient, in addition, a tumor sample at the diagnosis of relapse was included. Clinical information on patients whose samples were used in this study is summarized in Additional file 3: Table S1. All patients were diagnosed with Ewing sarcoma during the years of 1990 through 2012 and treated according to the protocols CESS 86, EICESS 92, EURO-Ewing 99 or Ewing 2008. Diagnosis of Ewing sarcoma was made based on histological appearance, cytogenetics and RT-PCR for *EWS-ETS* fusion; all tumors were reviewed by a reference pathologist within the EURO-Ewing study. No extraosseus tumors were included. All patients were treated at the University Hospital Freiburg. The investigations performed are in compliance with the Helsinki Declaration. Informed consent was obtained from all patients or their legal guardians and the analysis was approved by the ethics committee of the University of Freiburg.

Due to limited availability of material, samples used for PCR studies were mostly from different patients than serum and immunohistochemistry samples. Biopsies used for PCR were shock frozen and stored at −196 °C in liquid nitrogen. Serum was stored at −80 °C prior to measurement of IL6 using the ELISA-Kit IMMULITE 2000 IL6, Siemens Medical Solutions Diagnostics, Eschborn, Germany.

### Immunohistochemistry

For immunohistochemical staining, 3 μm sections of formalin fixed, paraffin embedded tissue samples were deparaffinzed by xylene and rehydrated by decreasing concentrations of ethanol. After heat induced antigen retrieval by pH9 Tris buffer (DAKO, Carpinteria, CA, USA), endogenous peroxidase activity was deactivated by 3 % hydrogen peroxide. Nonspecific protein binding sites were blocked by Protein Block (DAKO, Carpinteria, CA, USA). αIL6 polyclonal rabbit antibody (Cat-No: ab662 Abcam, Cambridge, UK) was incubated with the slides for 60 min. For detection, the polymer-based Envision Kit by DAKO (Carpinteria, CA, USA) was applied, including a secondary antibody and DAB (diaminobenzidine) for staining. After counterstain by hematoxylin, dehydration and coverslipping, stained sections were evaluated and digitized for histological photographs and quantification of staining (Hamamatsu NanoZoomer 2.0 HT, Hamamatsu Photonics, Hersching am Ammersee, Germany). Scoring of immunohistochemical staining for IL6, vimentin and smooth muscle antigen (SMA) was done as follows. Samples were scored as positive (+) in cases of intermedium/strong staining in more than 50 % of tissue or cell content. In addition, staining for IL6 was quantitatively analyzed by the area of stained cells/extracellular space and semi quantitatively by the intensity of staining. Distribution of staining was evaluated on the entire tumor section using a 20× lens. The percentage of positive cells/extracellular space within various fields was determined, and a mean score was calculated. The intensity was scored as no signal (0), weak signal (1), or intermedium (2) to strong signal (3). (Additional file 4: Table S2).

### Cell lines and culture

Seven cell lines were used in this study. The ES cell lines A4573, TC71, TC32, SK-N-MC, CHP-100 and JR were kindly provided by Jeff Toretsky (Georgetown University, Washington D.C., USA). Biological characteristics of these lines have been described earlier [27]. The cell line NK, positive for the *EWS/FLI1* fusion, has been newly derived in our laboratory from the tumor of a patient with metastatic ES. The IL6 negative prostate cancer cell line LNCaP was a gift from Eric Metzler (Department of Experimental Urology, University Hospital, Freiburg).

Conditions of cell culture included RPMI media supplemented with 10 % fetal calf serum (FCS), 100,000 IU/ml Penicilline, 100 μg/ml Streptomycine, temperature of 37 °C and 5 % CO2 atmosphere in the incubator. Condition of serum starvation as experimental setting was induced by medium change to medium without supplements 24 h after seeding.

### Reagents

Recombinant human IL6 (rhIL6, Cat. No 206-IL) and human anti-IL6R antibody (clone 17506, Cat. No MAB227) were purchased from R&D Systems, Minneapolis, USA. Antibodies used for immunoblot included rabbit anti-phospho-STAT3 polyclonal antibody (Cat. No 9131) and rabbit anti-STAT3 polyclonal antibody(Cat. No 9132), Cell Signaling TECHNOLOGY®, Frankfurt, Germany, anti-β-actin monoclonal antibody, clone AC-15, Sigma Aldrich, Munich, Germany, goat anti-mouse IgG-HRP and goat anti-rabbit IgG-HRP, Santa Cruz Biotechnology, Heidelberg, Germany. The mouse anti-γ1 PE, clone X40, BD Biosciences, Erembodegem, Belgium, mouse anti-human IL6R-phycoerythrin, clone 17506, R&D Systems, Minneapolis, USA and mouse anti-human CD130 PE, clone AM64, BD Pharmingen, San Diego, USA were used for flow cytometric analyses.

### RT-PCR

The following primers, synthesized by Eurofins MWG Synthesis GmbH, Ebersberg, Germany, were used in PCR reactions as published previously: IL6 [28], IL6ST [29], IL6R [30] and GAPDH [31]. Primer sequences are summarized in Additional file 5: Table S3. For RT-PCR, RNA extraction was performed using TRIzol, Invitrogen, Carlsbad, USA, and cDNA synthesis was done using the quantiscript protocol, Qiagen, Hilden, Germany. Primer annealing temperatures and cycling parameters were established individually for each gene of interest.

### Immunoblot

Immunoblot was performed as described before [32]. The first antibody (dilution 1:1,000 in PBS and 5 % bovine serum albumin) was incubated at 4 °C for 12 h. After washing the membrane three times, a secondary antibody was added for 30 min at room temperature, dilution 1:10,000 in PBS and 5 % BSA. Washing was repeated three times. Then, the protein of interest was made visible by chemoluminescence using ECL, Amersham/GE Healthcare Europe, Freiburg, Germany.

### Flow cytometric analysis of cell surface receptor expression

Staining of cell surface receptors was performed as described before [33]. In brief, cells were mechanically removed by gentle pipetting, washed twice and resuspended in cold (4 °C) PBS followed by an incubation with anti-human IL6R-phycoerythrin, anti-human CD130 PE and mouse anti-γ1 PE (30 min, 4 °C, dark room). A minimum of 10,000 cells was analyzed for IL6R and IL6ST cell surface receptor expression using FACScan, Becton Dickinson, Heidelberg, Germany.

### 3-(4,5 dimethylthiazol-2yl)-2,5-diphenyltetrazolium (MTT-) Assay

Cells were seeded in 96-well plates in medium with supplements as stated above, 5,000 cells/well, 100 μl/well and incubated overnight. 24 h afterward, medium was changed to medium without supplements. IL6 (10 ng/ml, 50 ng/ml and 100 ng/ml) was added on days 0 or 0, 2 and 4, respectively. The number of viable cells was inferred on days 3, 4 and 5 from the tetrazolium-reducing activity of mitochondrial dehydrogenases using the MTT-assay as described before [32]. Absorbance was measured at 570 and 620 nm using the spectra sunrise absorbance reader, Tecan, Männedorf, Switzerland. Background subtracted absorbance (i.e. 570 nm - 620 nm) was plotted as function of treatment.

### Apoptosis stain with propidium iodide

Cells were plated and treated as specified in the MTT section. On day 5, cells were harvested, centrifuged (1,000 rpm, 4 °C, 5 min) and washed with cold PBS. After another centrifugation step (1,000 rpm, 4 °C, 5 min) cells were incubated at 4 °C in hypotonic propidium iodide buffer as described before [32]. DNA length with a focus on the sub G1 section was measured using FACScan (Becton Dickinson, Heidelberg, Germany). 10,000 cells were measured per sample.

### CAM-Assay

2.5 × 10^6^ cells of ES cell line A4573, engineered to stably overexpress green fluorescent protein (i.e. A4573/GFP), where initially inoculated on the chorioallantoic membrane (CAM) of *ex ovo* prepared live chick embryos on day 9 (d9) of development (see reference [34] for details of egg preparation and CAM assay). On d10 three explants, each growing on a separate embryo, received in their immediate vicinity a bolus of 10 μl of a) PBS (control), b) 25 ng/μl IL6 (250 ng IL6) and c) 50 ng/μl IL6 (500 ng IL6), which was dissolved in PBS. On d14 A4573/GFP cells were quantitated under a fluorescent microscope as function of migration distance from the perimeter of the explant. For that purpose three images per tumor were taken, each superimposed by a 15 **×** 12 square grid that allowed to subdivide the entire image into five equal-size zones (each with 3 × 12 square area): zones 5 + 4 (= tumor core), 3 (tumor edge), 2 (near migration) and 1 (far migration). Cell coverage in each square was estimated for zones 3, 2 and 1 with values ranging from 0 (no cells present) to 5 (square 100 % filled with cells). Mean (± SEM) zone 1–3 coverage values of n = 3 independent experiments were computed for all three images per tumor as a measure of cell motility in response to PBS, 250 ng and 500 ng IL6.

### IL6 Elisa

For detection of IL6 in the supernatant cells were plated in 24-well plates at a concentration of 100,000 cells/well in 500 μl medium without supplements and incubated for 48 h. After centrifugation of the supernatant (1,000 rpm, 4 °C, 5 min) IL6 was measured using the ELISA-Kit IMMULITE 2000 IL6, Siemens Medical Solutions Diagnostics, Eschborn, Germany. The lowest detection level was 2 pg/ml.

### Statistics

Statistical analysis was performed using SPSS for Windows 20 (IBM Corp., Armonk, New York, USA). For statistical analysis of MTT and flow cytometry box plots were used to describe the distribution of the data. Since normal distribution could not be shown, median values and ranges were reported and nonparametric statistics were used to test differences among variables (Kruskal-Wallis rank test with adjacent post-hoc Mann–Whitney-*U*-Test). For CAM assay statistical differences among mean coverages of IL6-treated vs. control (PBS) treated explants were tested with non-parametric Wilcoxon rank sum tests.

All p-values were 2-sided and values equal or less than 0.05 were considered statistically significant. P values greater than 0.1 were reported as non-significant.

## Results

### A subgroup of ES patients shows elevated IL6 serum levels at diagnosis

IL6 serum levels were analyzed by ELISA in 12 ES patients at the time of diagnosis. IL6 levels above the lower detection limit of the assay could be demonstrated in all of them. Interestingly, a group of four patients with IL6 levels ≥ 20 pg/ml had a different clinical course than the other eight patients with lower levels (Table 1). Two of the four patients with higher IL6 levels had fever at diagnosis, and three of these four patients metastases at first diagnosis; all four patients died of their disease. In contrast, only two of the eight patients with IL6 levels below 20 pg/ml had fever, metastases and died of their disease.

**Table 1:** Serum IL6 levels at initial diagnosis of ES in 12 patients measured by ELISA

	IL6 ≥ 20 pg/ml	IL6 < 20 pg/ml
Patients	4	8
Fever at diagnosis	2	2
Metastasis at diagnosis	3	2
Death, due to disease progression	4	3
IL6-positive tumor (RT-PCR or IHC)	2/3	1/5

### ES tumors and cell lines express the IL6 receptor complex, IL6R and IL6ST

**Fig. 1:**
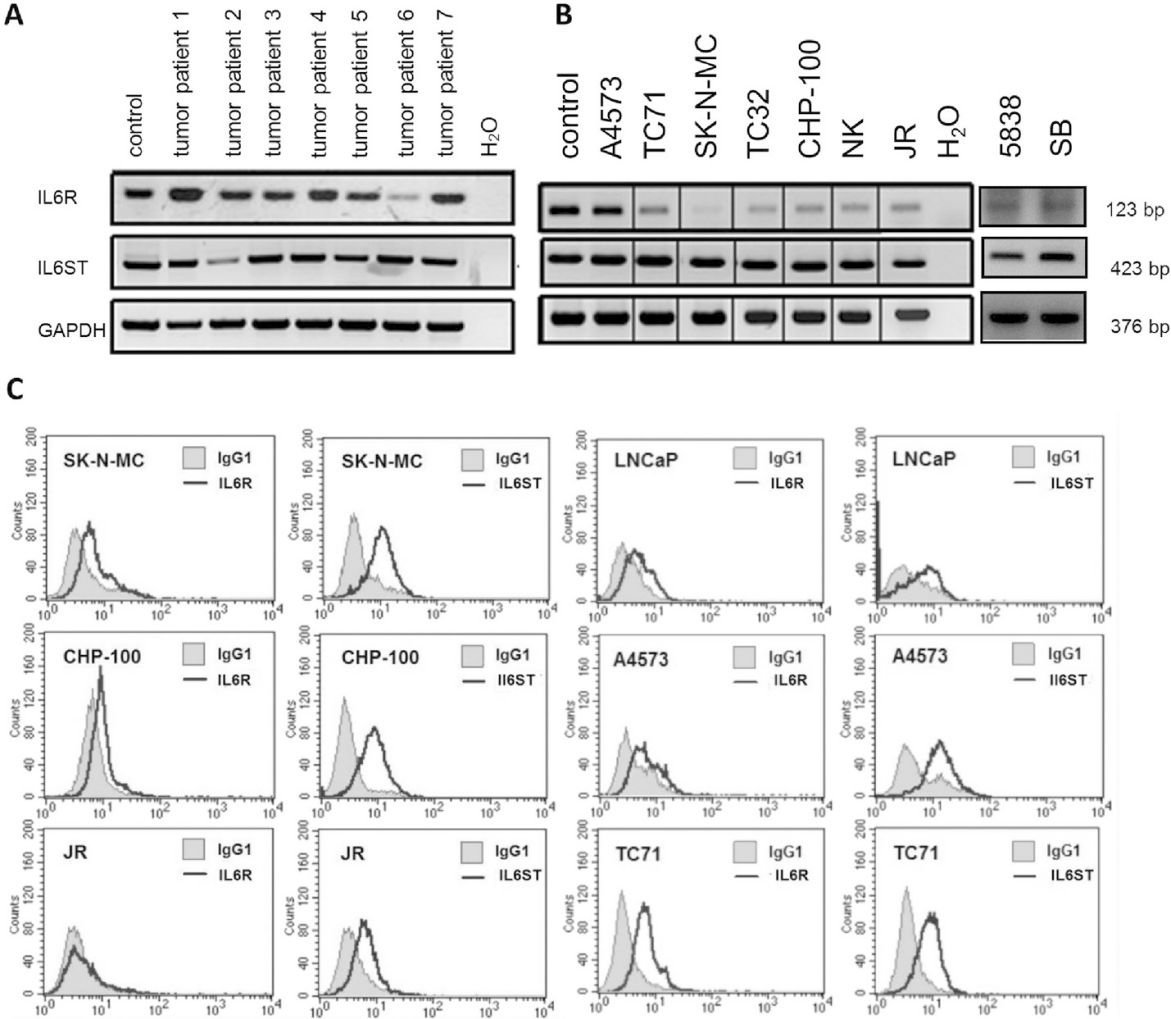
**a** IL6R and IL6ST mRNA expression of seven ES tumor samples by RT-PCR. IFN-γ treated PBMC were used as positive control (left lane). All tumor specimens expressed both IL6 receptor subunits. **b** IL6R and IL6ST mRNA expression in nine ES cell lines (RT-PCR). IFN-γ treated PBMC were used as positive controls (left lane). **c** Assessment of IL6 receptor complex cell surface expression by flow cytometry. The prostate cancer cell line LNCaP was used as a positive control. IL6R and IL6ST cell surface staining of 4 additional ES cell lines is shown in Additional file 1: Figure S1

The IL6 receptor complex is composed of two subunits: IL6R, the IL6 interacting part, and IL6ST, which interacts with the IL6R/IL6 complex. IL6ST mediates intracellular signal transduction. Applying RT-PCR to 7 tumor specimens we were able to show pronounced expression of both subunits in most tumor biopsies (Fig. 1a).

Analysis of the expression of both subunits in cell lines gave similar results (Fig. 1b). All ES cell lines showed constitutively high level IL6ST- and variable IL6R expression. Maximal IL6R abundance was found in the cell line A4573, lowest levels in SK-N-MC cells.

Flow cytometric analysis was applied to investigate whether IL6R and IL6ST were expressed on the cell surface of ES cells. High surface expression of IL6ST was noted for all cell lines. In line with the mRNA expression pattern, IL6R protein occurred at lower and variable levels, and was not detectable in cell line JR. The prostate cancer cell line LNCaP, used as an IL6R positive control, had similar expression levels of IL6R and IL6ST compared to ES cell lines (Fig. 1c and Additional file 1: Figure S1).

### The IL6 receptor complex is functional in ES cell lines

**Fig. 2:**
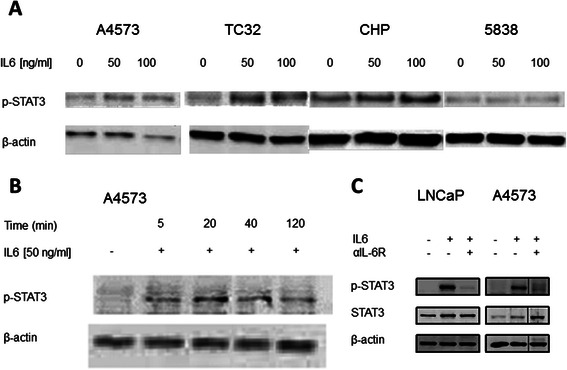
**a** p-STAT3 phospho-specific immunoblot of four ES cell lines. rhIL6-mediated STAT3 activation was detected after 20 min incubation of indicated IL6 concentrations in cell lines A4573, TC32 and CHP. All cell lines showed low intrinsic phosphorylation of STAT3. Cell line 5838 did not display an increase in p-STAT3 after incubation with rhIL6. **b** p-STAT3 phospho-specific immunoblot of cell line A4573 after incubation with 50 ng/ml rhIL6 for indicated time periods. Maximal levels of phosphorylated STAT3 were seen 20 min after adding rhIL6. STAT3 phosphorylation decreased after 2 h of incubation. **c** Phosphorylation of STAT3 in A4573 ES cell line is IL6-specific. Cells were stimulated with rhIL6 (100 ng/ml for 20 min). An IL6 receptor-specific antagonistic antibody (2 μg/ml) was added 30 min prior to rhIL6 incubation where indicated. The prostate cancer cell line LNCaP was used as a positive control. STAT3 phosphorylation mediated by rhIL6 could be blocked by an anti-IL6 receptor antibody. Data representative for three independent protein harvests and immunoblots

Interaction of IL6 with IL6R and subsequent formation of the heterodimer with IL6ST leads to phosphorylation of STAT3. To analyze the functionality of the IL6 receptor complex in ES cells, we performed STAT3 phospho-immunoblots of 8 ES cell lines (Fig. 2a and data not shown). In three cell lines – A4573, TC32 and CHP-100 – incubation with rhIL6 led to Tyr705-phosphorylation of STAT3 at IL6 concentrations as low as 50 ng/ml. All cell lines revealed low intrinsic activation of STAT3. Treatment of cell lines 5838, SB and JR with rhIL6 did not have any effect on STAT3 phosphorylation (Fig. 2a and data not shown). Regarding the time course of STAT3 phosphorylation after incubation with rhIL6, A4573 cells showed increased phosphorylation of STAT3 as early as 5 min after treatment with rhIL6, and maximal levels after 20 – 40 min. After 2 h STAT3 activation was reduced but still detectable (Fig. 2b).

Phosphorylation of STAT3 could be attributed to a functional IL6 receptor complex, since interference of the IL6R/IL6 interaction by an anti-IL6R-antibody, applied 30 min prior to treatment of the cells with rhIL6, completely inhibited STAT3 phosphorylation in both LNCaP positive control and A4573 ES cells. Consistent with the lack of protein expression of IL6R on the cell surface (Fig. 2c and data not shown), no STAT3 phosphorylation was detected in cell line JR after incubation with rhIL6.

### IL6 rescues ES cells from apoptosis during serum starvation

Since IL6 is known to stimulate proliferation and to inhibit apoptosis in various tumors, we first examined the influence of rhIL6 on cell growth in the ES cell line A4573. The functional IL6 receptor complex in A4573 cells triggers phosphorylation of STAT3 once cells are subjected to IL6. Judged by the number of viable cells 24 and 48 h post stimulation by MTT assay, however, a single bolus of rhIL6 was ineffective to induce proliferation of cells kept in serum-free medium (data not shown). As a consequence we decided to stimulate cell proliferation through repeated rhIL6 applications to aim for IL6 concentrations that remain more or less elevated over an extended time period mimicking more physiologic conditions.

**Fig. 3:**
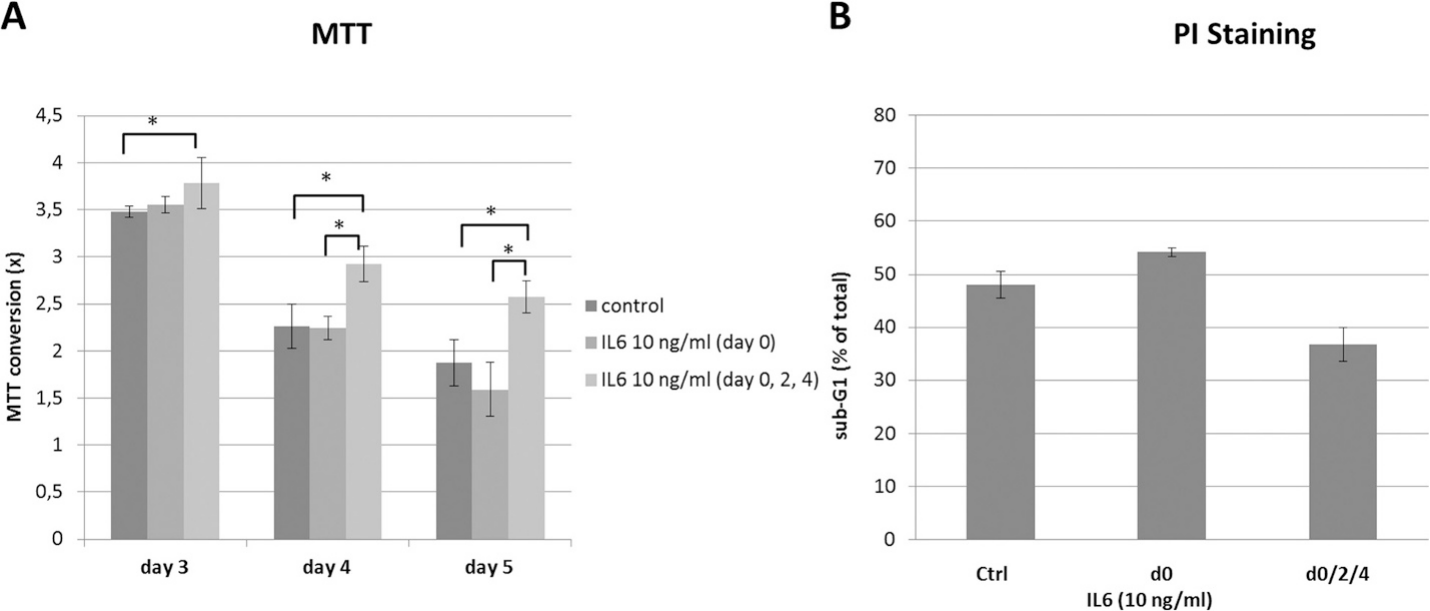
**a** Increased viability of serum-starved A4573 ES cells by repetitive stimulation with rhIL6 (d 0, 2, 4; 10 ng/ml). Fold-change (x) of MTT conversion rates (n = 5; mean ± SD) relative to rates of cells measured 24 h after seeding and set to 1 (100 %) are shown. Statistical significance of IL6 treatment relative to controls is indicated (Mann–Whitney-*U*-Test; * = p < 0.05). **b** Serial supply of rhIL6 reduces rate of apoptosis from serum starvation in A4573 ES cells. Serum-starved cells of ES cell line A4573 cells were stimulated repetitively with rhIL6 (10 ng/ml on indicated days) and harvested on day 5. Sub-G1 DNA content was used as a measure of apoptosis. Bars (n = 3; mean ± SD) indicate fraction of cells (%) containing a sub-G1 DNA content. Similar results could be shown in two other independent experiments

Cells of all subgroups (untreated controls, single-bolus IL6 and repetitively treated with IL6) continued to grow until day 3, followed by a decline in viable cells on day 4 and further on day 5. Stimulating serum-starved A4573 cells through repetitive applications of rhIL6 (d0, d2, d4 at 10 ng/ml) yielded, compared to untreated (control) or single-bolus treated (d0) cells, a slight increase in the number of viable cells on day 3 and a significantly smaller decline in the fraction of viable cells on days four and five post-stimulation as indicated in Fig. 3a. To further characterize the cause for the improved viability of serum-deprived yet serially IL6 exposed cells, we analyzed the sub-G1 content of cells by flow cytometry. According to these data, serial treatment of ES cells with rhIL6 inhibited apoptosis, in turn yielding a higher number of viable A4573 cells (Fig. 3b).

### IL6 promotes migration of ES cells

**Fig. 4:**
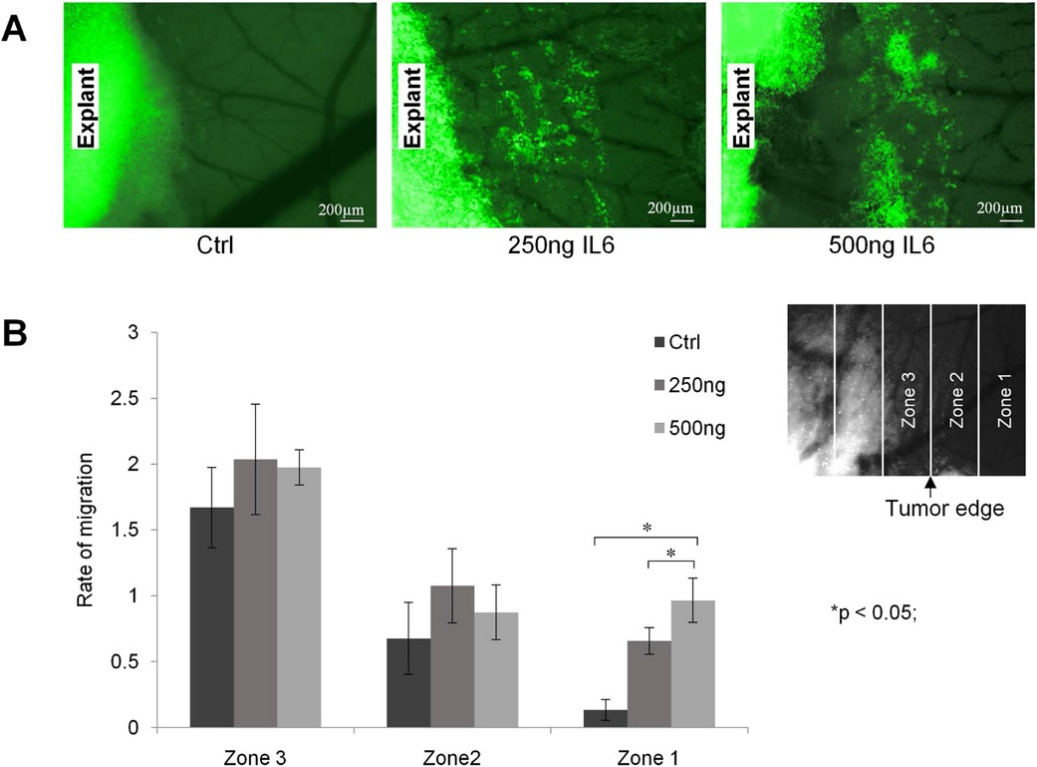
**a** Immunofluorescence of A4573/GFP ES cells grown on a CAM and treated with single IL6 dose as indicated. **b** Cell coverage across equal-size zones 3, 2 and 1 (right) was estimated by superimposed 3 × 12 square area with values ranging from 0 (no cells present) to 5 (square 100 % filled with cells). See Methods for details. The bar graph (left) illustrates relative mean (± SEM) coverage values for zones 3, 2 and 1 from a total of three independent experiments as a measure of cell motility in response to same-volume applications of PBS, 250 ng and 500 ng rhIL6. Addition of rhIL6 dose-dependently and significantly (* = p < 0.05) increased motility of A4573 ES cells on the surface of the CAM from zone 3 (explant perimeter) to zone 1 (furthest distance)

Since IL6 promotes cell dissemination in a great variety of tumors we thus analyzed migration of A4573/GFP ES tumor cells in CAM assays. As depicted in Fig. 4, single high-dose rhIL6 application was sufficient in triggering a higher degree of cell migration into regions distal from the primary tumor (zone 1) compared to PBS-treated controls. We found a significant increase in cell dissemination using 250 ng/ml and 500 ng/ml rhIL6, relative to a PBS load of equal volume. Moreover, 500 ng/ml rhIL6 increased migration into zone 1 to a larger extent than the lower rhIL6 dose.

### IL6 is expressed by cells of the tumor microenvironment

To gain deeper insight into the origin of IL6 production in ES patients, we analyzed the expression of IL6 mRNA and protein in ES cell lines and their supernatants as well as in biopsy specimens of primary tumors.

**Fig. 5:**
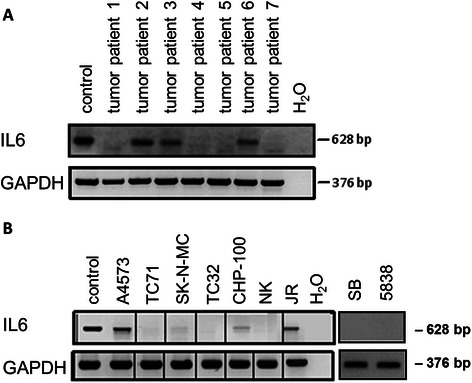
**a** Detection of IL6 mRNA expression in 7 ES tumor samples using RT-PCR. IFN-γ treated PBMC were used as positive control. 3 out of 7 tumor samples expressed IL6 mRNA. **b** IL6 mRNA expression in 9 ES cell lines by RT-PCR. IFN-γ treated PBMC were used as positive control. 4 out of 9 cell lines expressed IL6 mRNA

Analysis of IL6 mRNA expression in 9 ES cell lines by RT-PCR showed detectable transcript levels in four of them, with cell lines A4573 and JR displaying the highest expression levels. CHP-100 and SK-N-MC lines demonstrated much lower levels (Fig. 5b). Except for CHP-100, IL6 mRNA positive cell lines secreted detectable concentrations of IL6 protein (ELISA detection) into the supernatant when incubated for 48 h in serum without supplements. Levels of secreted IL6 in the supernatant were low, except for cell line JR (Table 2).

**Table 2:** Concentration of IL6 in the supernatant of 5 ES cell lines cultured *in vitro* (ELISA)

ES cell line	IL6 concentration (pg/ml)
A4573	14.43 ± 0.15
TC71	<2
SK-N-MC	2.50 ± 0.44
CHP-100	<2
JR	279.67 ± 24.70

Similar to ES cell lines, of which 4 out of 9 expressed mRNA for IL6, only three of seven tumor biopsies expressed mRNA for IL6 (Fig. 5a). This is in contrast to the expression of the IL6 receptor subunits, which were found in all cell lines and tumor samples. Consequently, the origin of IL6 in serum of patients with Ewing sarcoma might not be attributed to the ES cells themselves in all instances.

**Fig. 6:**
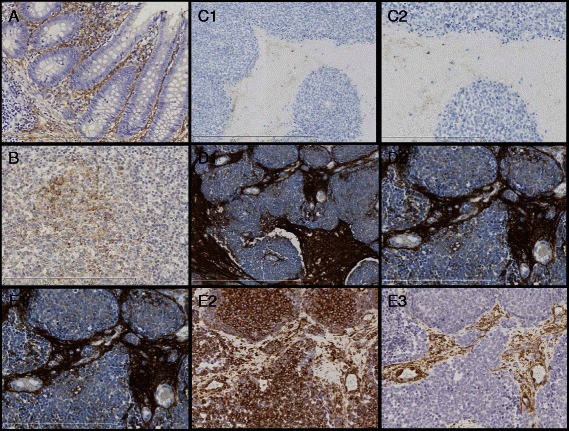
Immunohistochemical analysis of two ES tumor specimens and control tissues for IL6, vimentin and smooth muscle actin (SMA). Pictures A and B show staining of control tissues with anti-IL6-ab (both 200×): **a** Appendix: positive staining of cells/extracellular space of lamina propria. **b** Tonsil: positive staining of cells/extracellular space in center of lymphoid follicles. Pictures C and D: examples of ES, negative and positive for IL6. **c1** and **c2** negative for IL6 in connective tissue septa within the tumor (Pt. 8). **d1** and **d2**) positive for IL6 in connective tissue septa within the tumor (Pt. 7). In both samples no staining could be observed in tumor tissue. **c1** and **d1**: 100×, **c2** and **d2**: 200×. Pictures **e1**-**e3**: comparison of different stainings of ES, positive case (Pt. 7), all 200×): **e1** IL6 staining, showing positivity in connective tissue septa between negative (blue) tumor cells. **e2** Staining against vimentin demonstrates mesenchymal character of both tumor cells and cells in connective tissue septa. **e3** Smooth muscle actin highlights the cells within the connective tissue septa, defining them as fibroblasts

To further examine possible sources of IL6 in ES we used an αIL6 antibody to stain tumor specimens from 4 ES patients. As demonstrated in Fig. 6 and Additional file 2: Figure S2, IL6 expression was found mainly in the intraseptal regions of tumor sections. By further immunohistochemical staining against vimentin and smooth muscle antigen (SMA), the cells in the septa were identified as fibroblasts by positivity for both markers. Within the septa no other significant cell population could be identified, except of very rare single lymphocytes and histiocytes. ES cells themselves were found to be IL6- negative. Comparing IL6 serum level with IL6 expression in ES tumor tissue by RT-PCR or immunohistochemistry in 8 patients, we found that two of three patients with IL6-positive tumors had IL6 serum level ≥ 20 pg/ml at diagnosis, whereas four of five patients with IL6-negative tumors had serum IL6 levels < 20 pg/ml (Table 1, Additional file 2: Figure S2). Consequently, the tumor microenvironment could be a source of IL6 detected in the serum of ES patients and paracrine mechanisms are possible to govern apoptotic or disseminating cell fates.

## Discussion

Our results demonstrate that the IL6 pathway is functional in ES cells. Treatment of ES cells *in vitro* and CAM grafts with rhIL6 inhibited apoptosis and supported cell dissemination. Evidence of IL6 expression in the tumor stroma of primary ES and dismal prognosis of patients presenting with higher IL6 levels at diagnosis further suggest a biological relevance of this cytokine in ES pathogenesis.

IL6 has a wide range of tumor promoting activities in a large variety of cancer cells. Its impact on malignant cells includes the stimulation of anti-apoptotic, proliferative and migratory activities, thereby enhancing tumor initiation and progression (reviewed in [6, 7]). Two years ago Blanchard et al. published the first investigation of the biological effects of different cytokines including IL6 in bone sarcoma cells. In their work, treatment with rhIL6 and sIL6R for 3 days in serum-depleted media (1 % FBS) enhanced proliferation in 10 different ES cell lines [35]. Though we did not observe enhancement of cell proliferation by IL6 in our serum-free system, IL6 protected cells from apoptosis through serum starvation. Observations of IL6 action under circumstances of cell stress in other tumor cell models support our results. In neuroblastoma, IL6 enhanced cell survival in cells cultivated in serum-deprived media [14, 36]. This protective effect was shown to be linked to the induction of F-thrombospondin [36].

Micrometastasis and systemic disease are one of the key features of ES [3]. Applying CAM assay we demonstrated a significant increase in ES cell dissemination into the CAM in response to single bolus treatment with a relatively high-dose of rhIL6. In contrast, non-treated cells, showing low-level intrinsic expression of IL6, had only scattered cells at more distant tumor sites. Our results are in concordance with data for breast cancer and glioblastoma showing IL6-dependent promotion of cell invasion into the basal membrane and CAM, respectively [37, 38].

Analyzing the IL6 signaling pathway we detected expression of receptor molecules IL6R and IL6ST by RT-PCR and flow cytometry in virtually all cell lines studied. This is similar to data for neuroblastoma showing expression of IL6R/IL6R mRNA in most of the cell lines studied [14]. RT-PCR analysis of primary ES tumors confirmed the expression pattern of cell lines with strong expression of IL6R and IL6ST in all tumor samples. Application of rhIL6 to ES cells led via the IL6R/IL6ST complex to STAT3 activation which is known to induce the expression of genes protecting from apoptosis and promoting migration.

In contrast to the expression of the IL6 receptor components in almost all ES cell lines and tumor samples, IL6 was found to be expressed in less than half of tumors applying RT-PCR. To explore the cellular origin of IL6 detected in the serum of ES patients, we performed immunohistochemistry on ES tumors. We found that a subgroup of ES tumors strongly expressed IL6 in the tumor connective tissue septa. Interestingly, these patients also had high IL6 serum levels indicating that IL6 secreted by ES tumor adjacent fibroblasts could be a major source for IL6 detected in the serum of ES patients. A similar immunohistochemical IL6 expression pattern has been described in primary colon carcinoma and invasive mammary carcinoma [39, 40]. Among other cell types, cancer-associated fibroblasts (CAF) stained positive for IL6 [6]. In addition, Rakan et al. demonstrated that CAF expressed IL6 and stimulated growth and invasiveness of breast cancer cells, emphasizing the role of the microenvironment [41].

Key biological features underlying the development of solid malignancies – uncontrolled cell division and growth, angiogenesis, invasion and metastasis – are all linked to inflammation [5]. Since it has been shown before that ES patients presenting with fever at diagnosis had a higher risk for metastases and death from their disease [9], and that IL6 is a main mediator of the febrile systemic response [42], we were intrigued to correlate IL6 serum levels with clinical data from ES patients. We found that 2 out of 4 patients with IL6 levels ≥ 20 pg/ml had fever and 3 out of 4 metastases at diagnosis, whereas only two out eight patients with IL6 levels below 20 pg/ml presented with fever and metastases at diagnosis. Though this patient cohort is too small for any statistical analysis, it indicates that IL6 might at least partly play a role in mediating effects such as fever and formation of metastases. This hypothesis is supported by results of a study by Rutkowski et al. who found elevated serum levels of IL6 to strongly correlate with tumor size, and inversely with OS and DFS in patients with malignant bone tumors, including patients with ES [10].

Due to the small group size, a prognostic impact of IL6 serum levels in ES patients at initial diagnosis on survival cannot be inferred from our data. Nevertheless, our data point to the need for a systematic analysis in a greater patient cohort aiming to elucidate the prognostic value of IL6 serum levels in ES patients.

## Conclusion

We were able to show a substantial contribution of rhIL6 in the inhibition of ES cell death during a period of cell stress. In particular, cells located in central, vessel-remote areas of the tumor are known to suffer from critical shortages of supplied nutrients and oxygen. IL6 might support mechanisms to overcome the hypoxic/ischemic challenge until vascularization and nutritional status improve. Furthermore, IL6 promotes cell dissemination to support evasion from poorly supplied tumor areas. Future studies should provide insights into the gene expression changes that are triggered in ES cells subjected to persistent rhIL6 exposure and required in yielding the anti-apoptotic and pro-disseminating ES responses.

## Additional files

Additional file 1: Figure S1. Cell surface staining for IL6R and IL6ST by flow cytometry in 4 additional ES cell lines. All cell lines express IL6R and IL6ST. (PPTX 172 kb)
Additional file 2: Figure S2. Two additional ES tumors demonstrating expression of IL6 in septa within tumor tissue (A: Pt. 9 and B: Pt. 10). Tumor cells were negative for IL6. Both specimens are from patients with high serum levels for IL6 (pt.9: 122 pg/ml and pt. 10: 139 pg/ml). (12,5× upper row, 200× lower row). (PPTX 1288 kb)
Additional file 3: **Table S1. a** Patient data for human tumor samples used. **Table S1. b** Patient data for human serum samples used. (DOCX 18 kb)
Additional file 4: Table S2. Immunohistochemical analysis of IL6, Vimentin and SMA in tumor tissues. (DOCX 15 kb)
Additional file 5: Table S3. Primer sequences for IL6, IL6ST, IL6R and GAPDH.(DOCX 13 kb)
