# Supplementary figures and images for: IL6 secreted by Ewing sarcoma tumor microenvironment confers anti-apoptotic and cell-disseminating paracrine responses in Ewing sarcoma cells
Source: BMC Cancer. 2015 Jul 28;15:552. doi: 10.1186/s12885-015-1564-7 (PMC4517368; doi:10.1186/s12885-015-1564-7)

## Slide 1
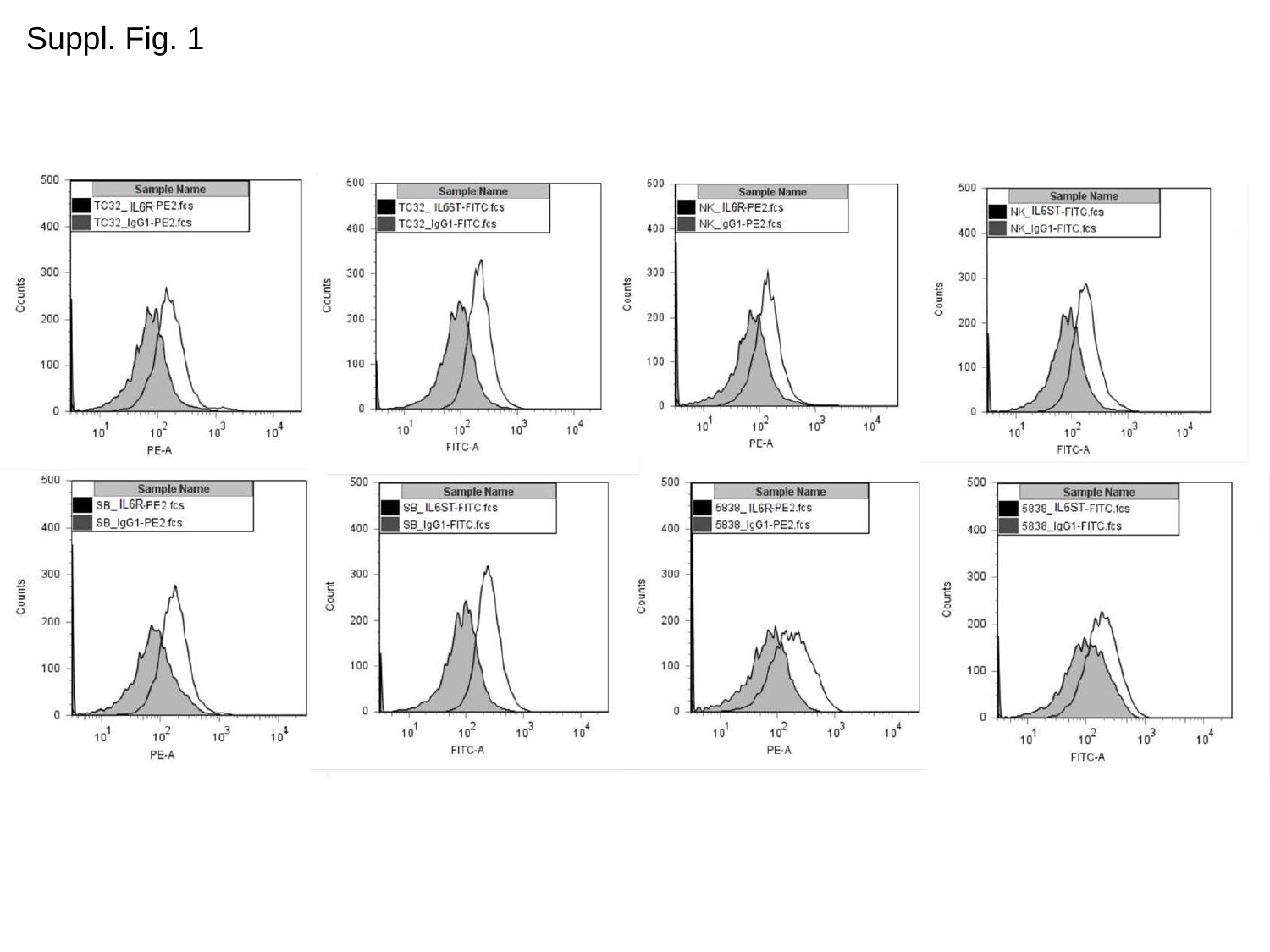

Suppl. Fig. 1

Supplement: Additional file 1: Figure S1. — Cell surface staining for IL6R and IL6ST by flow cytometry in 4 additional ES cell lines. All cell lines express IL6R and IL6ST. (PPTX 172 kb) [file 12885_2015_1564_MOESM1_ESM.pptx]

## Slide 1
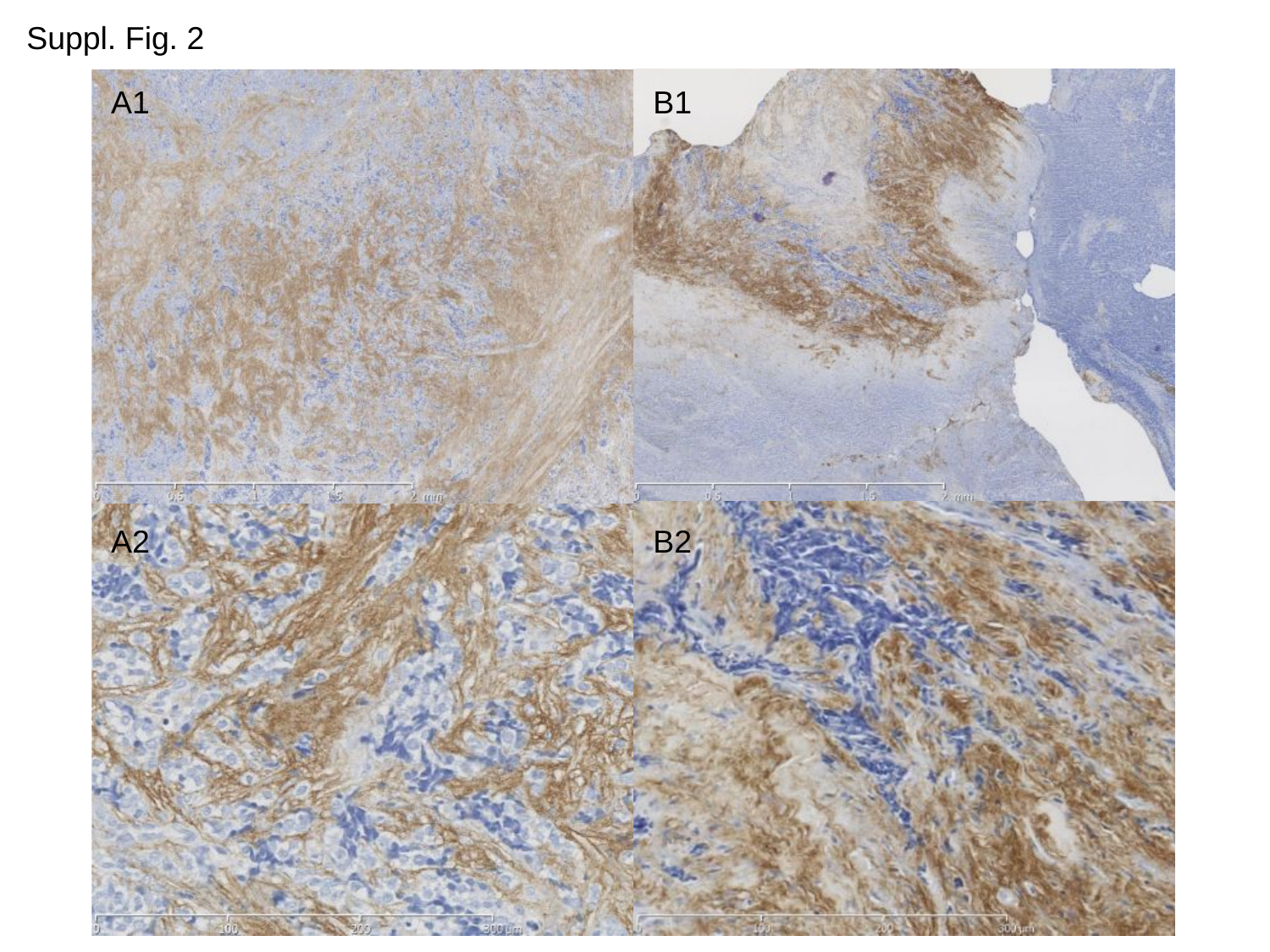

Suppl. Fig. 2
A1
B1
A2
B2

Supplement: Additional file 2: Figure S2. — Two additional ES tumors demonstrating expression of IL6 in septa within tumor tissue (A: Pt. 9 and B: Pt. 10). Tumor cells were negative for IL6. Both specimens are from patients with high serum levels for IL6 (pt.9: 122 pg/ml and pt. 10: 139 pg/ml). (12,5× upper row, 200× lower row). (PPTX 1288 kb) [file 12885_2015_1564_MOESM2_ESM.pptx]
