# Supplementary material for: IL6 secreted by Ewing sarcoma tumor microenvironment confers anti-apoptotic and cell-disseminating paracrine responses in Ewing sarcoma cells
Source: BMC Cancer. 2015 Jul 28;15:552. doi: 10.1186/s12885-015-1564-7 (PMC4517368; doi:10.1186/s12885-015-1564-7)
Supplement: Additional file 3: — Table S1. a Patient data for human tumor samples used. Table S1. b Patient data for human serum samples used. (DOCX 18 kb) [file 12885_2015_1564_MOESM3_ESM.docx]

**Supplemental Table 1a** Patient data for human tumor samples used

| Pt. | Sample | Location | Age (y) | Sex | Met.  at dx. | Surgery / Site | Timepoint of surgery |
| --- | --- | --- | --- | --- | --- | --- | --- |
| 1 | PCR 1 | 11^th^ rib | 17 | M | n | Bx, primary tumor | Initial dx, pre-chemo |
| 2 | PCR 2 | 9^th^ rib | 8 | M | y | Bx, primary tumor | Initial dx, pre-chemo |
| 3 | PCR 3 | Prox. tibia | 19 | M | n | Bx, primary tumor | Initial dx, pre-chemo |
| 4 | PCR 4 | Left humerus | 9 | F | y | Bx, primary tumor | Initial dx, pre-chemo |
| 5 | PCR 5 | Sacrum | 16 | F | n | Bx, primary tumor | Initial dx, pre-chemo |
| 6 | PCR 6 | Scapula | 13 | F | n | Bx, primary tumor | Initial dx, pre-chemo |
| 7 | PCR 7 | 3^rd^ rib right | 8 | F | n | Bx, primary tumor | Initial dx, pre-chemo |
| 7 | Hist. 1 | 9^th^ rib left | 13 | F |  | Bx, metastasis | 1. Relapse, pre-chemo |
| 8 | Hist. 2 | Sacrum | 16 | M | y | Bx, primary tumor | Initial dx, pre-chemo |
| 9 | Hist. 3 | Iliac bone | 14 | F | y | Bx, primary tumor | Initial dx, pre-chemo |
| 10 | Hist. 4 | 2^nd^ rib | 17 | F | n | Bx, primary tumor | Initial dx, pre-chemo |

Patient 7 had a relapse located at the 9^th^ rib, 5 years after diagnosis

**Supplemental Table 1b** Patient data for human serum samples used

| Pt. | Serum Sample | Location | Age (y) | Sex | Met.  at dx. | Timepoint of serum draw |
| --- | --- | --- | --- | --- | --- | --- |
| 1 | 1 | 11^th^ rib | 17 | M | n | Initial dx, pre-chemo |
| 2 | 2 | 9^th^ rib | 8 | M | y | Initial dx, pre-chemo |
| 4 | 3 | Left humerus | 9 | F | y | Initial dx, pre-chemo |
| 5 | 4 | Sacrum | 16 | F | n | Initial dx, pre-chemo |
| 7 | 5 | 3^rd^ rib right | 8 | F | n | Initial dx, pre-chemo |
| 8 | 6 | Sacrum | 16 | M | y | Initial dx, pre-chemo |
| 9 | 7 | Iliac bone | 14 | F | y | Initial dx, pre-chemo |
| 10 | 8 | 2^nd^ rib | 17 | F | n | Initial dx, pre-chemo |
| 11 | 9 | Tibia | 8 | M | n | Initial dx, pre-chemo |
| 12 | 10 | Tibia | 16 | M | y | Initial dx, pre-chemo |
| 13 | 11 | Calcaneus | 10 | M | n | Initial dx, pre-chemo |
| 14 | 12 | 7^th^ rib | 17 | F | n | Initial dx, pre-chemo |
