# Supplementary material for: IL6 secreted by Ewing sarcoma tumor microenvironment confers anti-apoptotic and cell-disseminating paracrine responses in Ewing sarcoma cells
Source: BMC Cancer. 2015 Jul 28;15:552. doi: 10.1186/s12885-015-1564-7 (PMC4517368; doi:10.1186/s12885-015-1564-7)
Supplement: Additional file 4: Table S2. — Immunohistochemical analysis of IL6, Vimentin and SMA in tumor tissues. (DOCX 15 kb) [file 12885_2015_1564_MOESM4_ESM.docx]

**Supplemental Table 2**

IL6 Fwd: ATG AAC TCC TTC TCC ACA AGC GC

IL6 Rev: GAA GAG CCC TCA GGC TGG ACT G

IL6ST Fwd: ACA GAT GAA GGT GGG AAG GAT

IL6ST Rev: AGA TGA CAT GCA TGA AGA CCC

IL6R Fwd: CCC ATC CCT GAC GAC AA

IL6R Rev ACT GCT AAC TGG CAG GAG AA

GAPDH Fwd: CCA TGA CAA CTT TGG TAT CGT GG

GAPDH Rev: GTC GCT GTT GAA GTC AGA GGA GAC
