# Supplementary material for: IL6 secreted by Ewing sarcoma tumor microenvironment confers anti-apoptotic and cell-disseminating paracrine responses in Ewing sarcoma cells
Source: BMC Cancer. 2015 Jul 28;15:552. doi: 10.1186/s12885-015-1564-7 (PMC4517368; doi:10.1186/s12885-015-1564-7)
Supplement: Additional file 5: Table S3. — Primer sequences for IL6, IL6ST, IL6R and GAPDH.(DOCX 13 kb) [file 12885_2015_1564_MOESM5_ESM.docx]

**Supplemental Table 3 Immunohistochemical analysis of IL6, Vimentin and SMA in tumor tissues**

| Patient | IL6 | IL6 pos. area | IL6 Intensity | | Vimentin | SMA |
| --- | --- | --- | --- | --- | --- | --- |
| 7 | t- ct + | 100% | 3+ | | t+,fb+ | t-,fb+ |
| 8 | t- ct + | 10% | | 1+-2+ | t+,fb+ | t-,fb+ |
| 9 | t- ct + | 50% | | 2+-3+ | t-,fb+ | t(+),fb+ |
| 10 | t- ct + | 60% | | 2+-3+ | t(+),fb+ | t(+),fb+ |

t, tumor; ct, connective tissue; fb, fibroblasts; -, negative staining; +, positive staining; intensity: 0-3, 3: maximum staining
